# Supplementary figures and images for: Template switching between the leading and lagging strands at replication forks generates inverted copy number variants through hairpin-capped extrachromosomal DNA
Source: PLoS Genet. 2024 Jan 4;20(1):e1010850. doi: 10.1371/journal.pgen.1010850 (PMC10766183; doi:10.1371/journal.pgen.1010850)

S1\_Fig

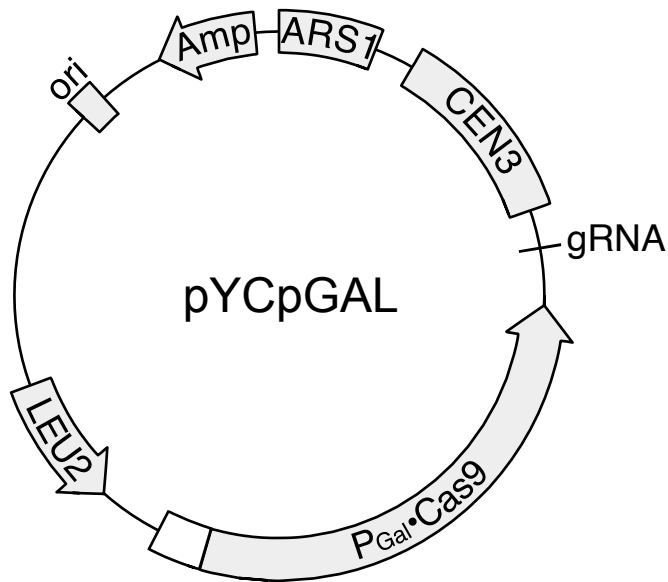

Supplement: S1 Fig — (PDF) [file pgen.1010850.s001.pdf]

S2\_Fig

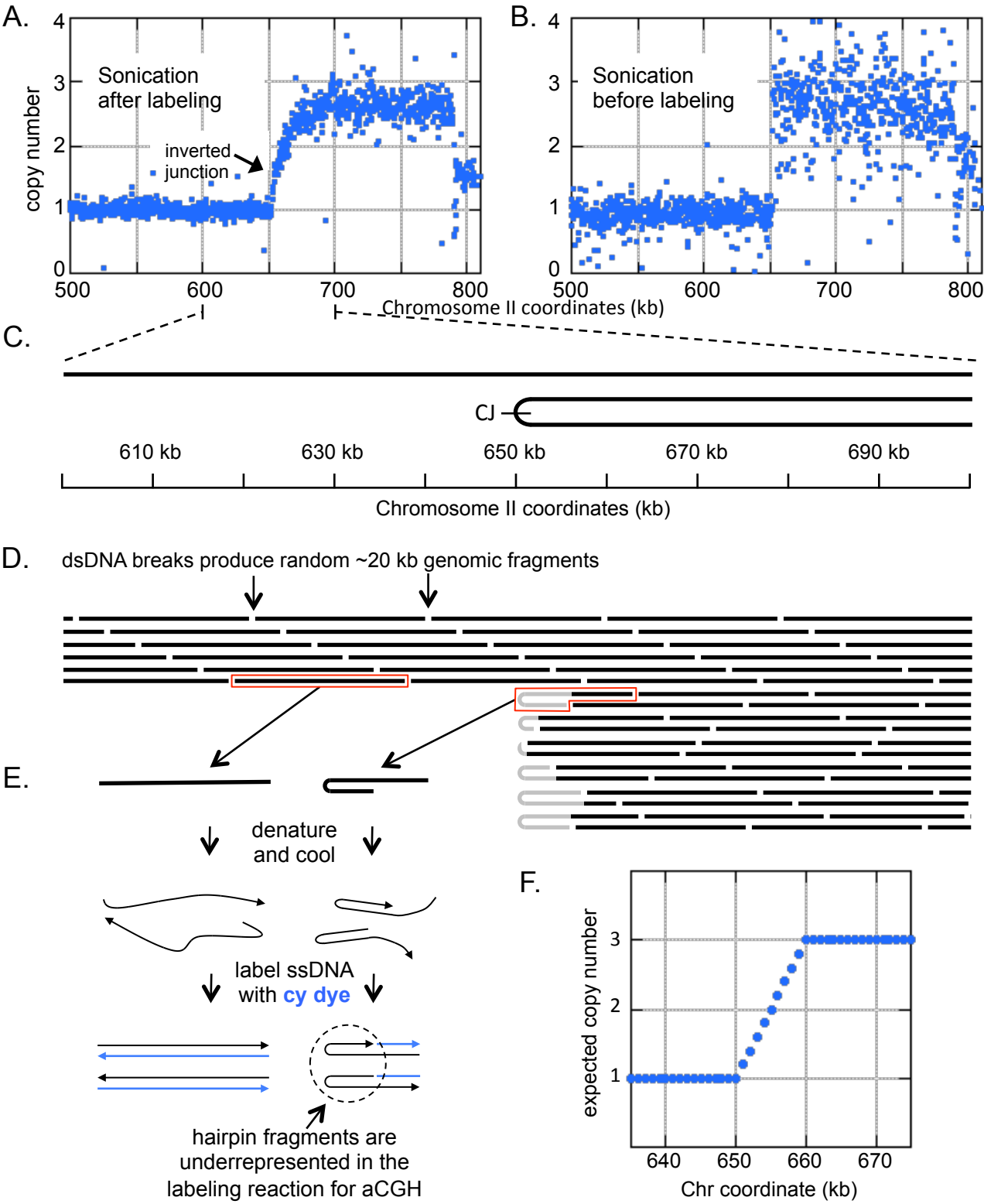

Supplement: S2 Fig — (A) aCGH of clone A1 shown for coordinates 500 to 813 kb. The DNA isolated for aCGH had an average size of ~20–40 kb. To label this DNA with Cy dyes, the DNA was denatured and random primers added for DNA polymerase to synthesize labeled strands. After labeling, the DNA was sonicated to ~500 bp and hybridized to an array. (B) The same DNA as in (A) was sonicated before denaturation and labeling. This method eliminated the gradual transition from 1 to 3 copies and produced an abrupt transition at the same site. (C) An illustration of the centromere proximal junction at ~650 kb of clone A1 with 50 bp of flanking genomic DNA. (D) DNA fragments of 20 kb are illustrated in a 3:1 proportion left and right of the centromere-proximal junction (CJ), respectively. (E) Representative molecules that either lack or contain the CJ palindrome. After denaturation and cooling the hairpins reform duplex DNA and are not available for priming and cy dye incorporation. Because breaks are in random places, different fragments will have different amounts of DNA excluded from the labeling reaction. These underrepresented regions are illustrated in gray in panel (D). (F) Quantification of the copy numbers expected across the site of the inversion junction generated from the schematic example in (D,E). Note that when the DNA was sheared to ~500 bp before labeling (B), the region up to within 250 bp of the inverted junction was now available for labeling with cy dyes, with a resulting clean discontinuity in the aCGH signal in place of the previous waterfall pattern. (PDF) [file pgen.1010850.s002.pdf]

S3\_Fig

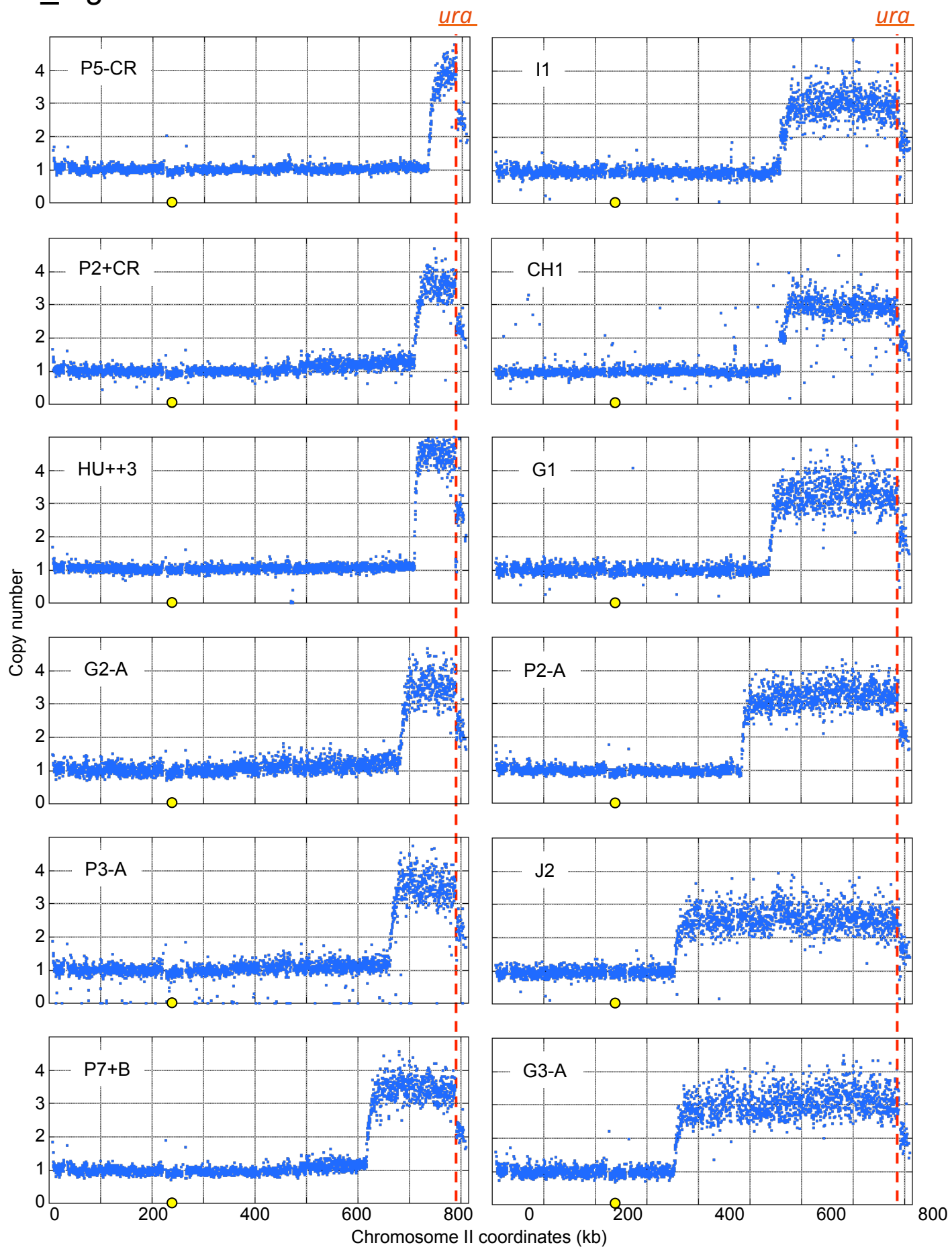

Supplement: S3 Fig — All chromosomes were recovered after growth in normal medium with the exception of the clone labeled HU++3 that was obtained from incubation in 200 mM hydroxyurea. Notice the different features in the right and left amplification junctions. The left junction shows a gradual change in copy number from 1 to ~ 3 copies and occurs at variable sites along chromosome II centromere proximal to SUL1. The left boundary of each inversion junction shows the characteristic “waterfall” transition between copy numbers. In contrast, the right junction is an abrupt change in copy number from ~3 to 2 copies created by recombination between ura and ra3 (on chromosome IX). The aCGH profiles of chromosome IX were identical to that shown in Fig 2B left. (PDF) [file pgen.1010850.s003.pdf]

S4\_Fig: Dicentric → centromere deletion

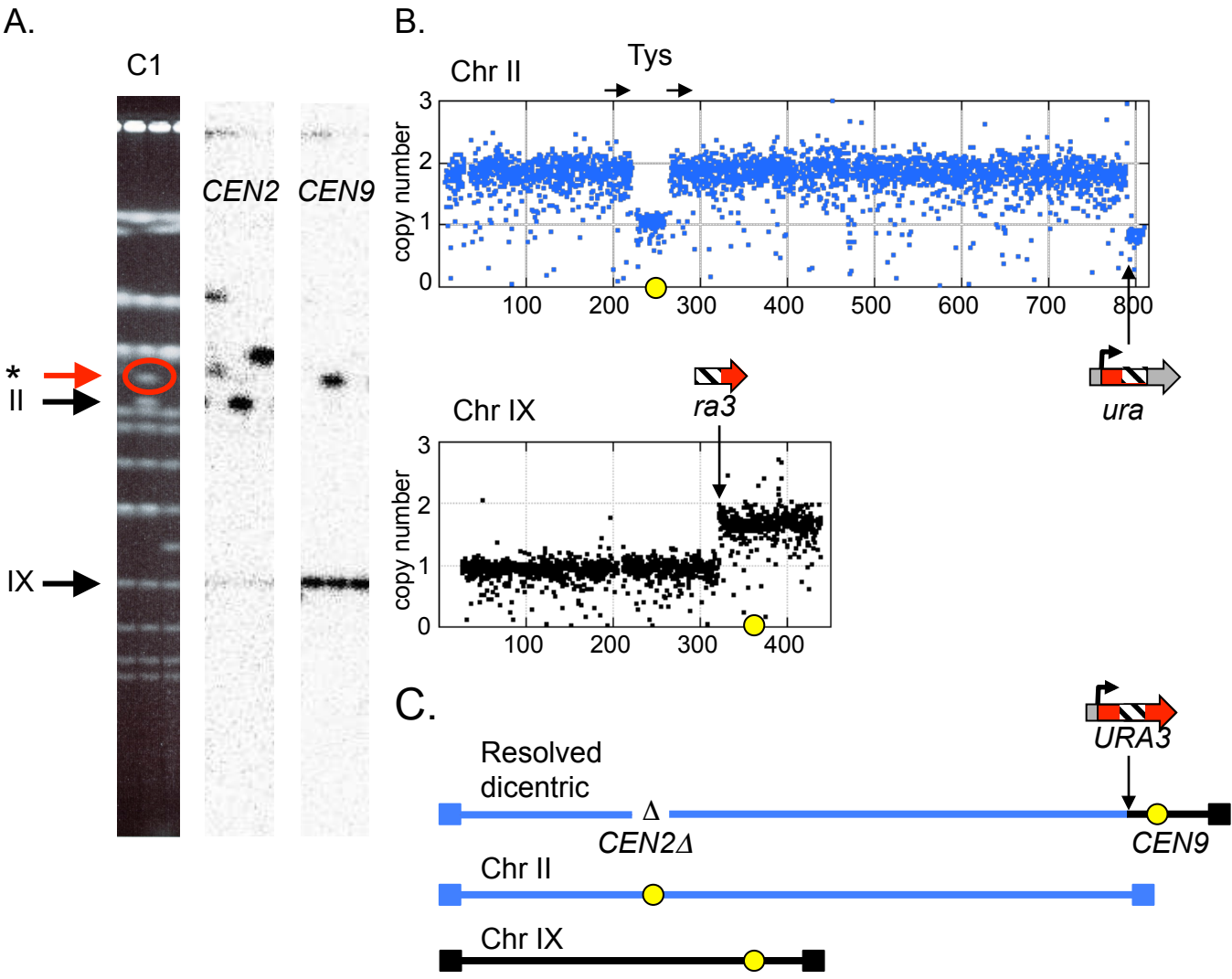

Supplement: S4 Fig — (A) Ethidium bromide stained gel of clone C1 (center lane) with a neochromosome that is larger than the native chromosome II. (The flanking gel lanes contained other Ura+ isolates.) Southern hybridizations indicate that the neochromosome has retained CEN9 and lost CEN2 and that there is an unaltered version chromosome II. (B) ArrayCGH confirms that most of chromosome II has been duplicated but one copy of the chromosome has lost its CEN2 sequence by recombination between the directly repeated Ty elements on either side of CEN2. The breaks in copy number on chromosome IX and the distal part of chromosome II mark the sites of the two URA3 fragments. (C) The most parsimonious organization of the duplicated parts of chromosomes II and IX produce a neochromosome that is consistent with the size estimated from the CHEF gel. (PDF) [file pgen.1010850.s004.pdf]

S5\_Fig: Dicentric → de novo telomere addition

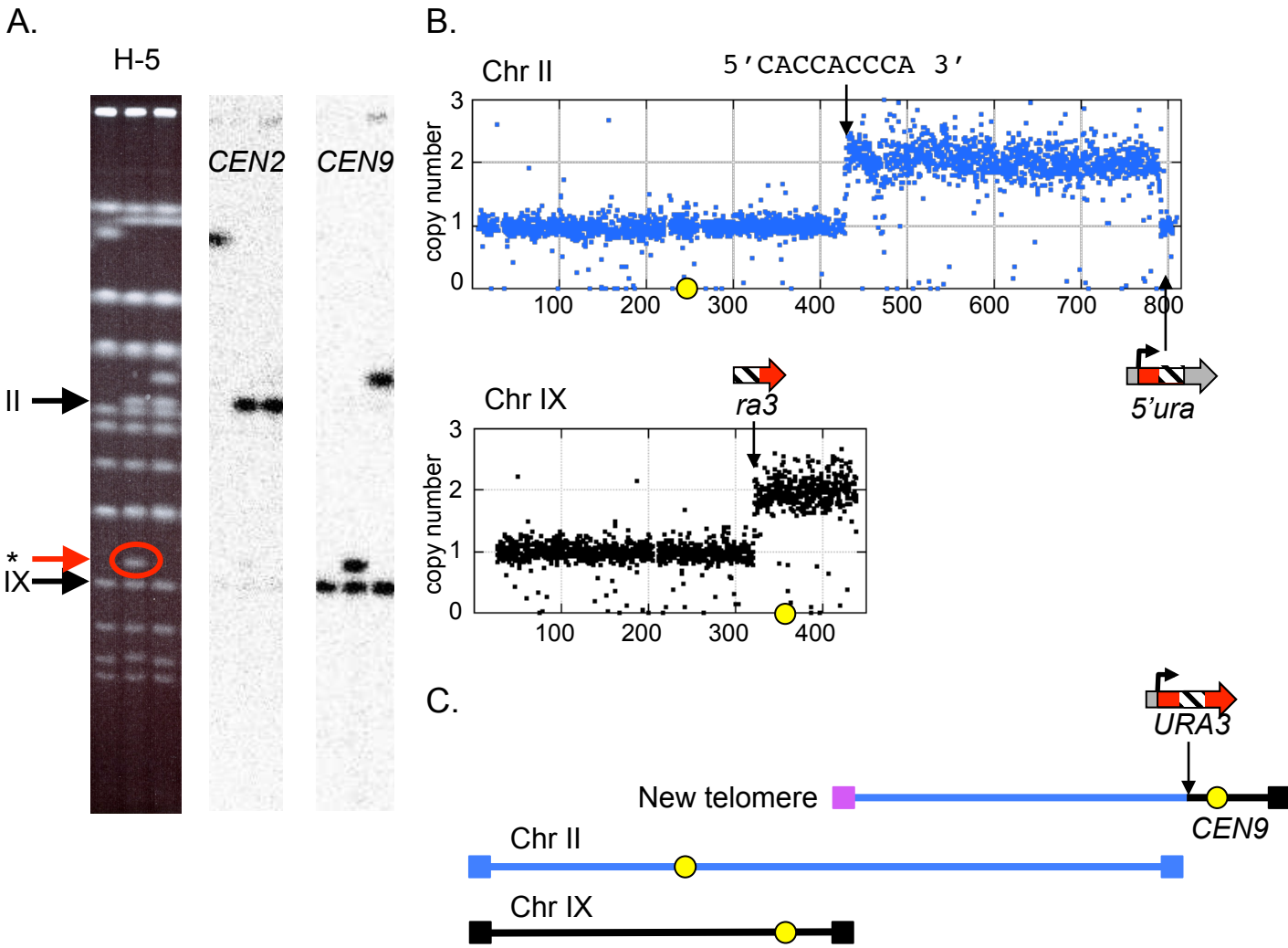

Supplement: S5 Fig — (A) Ethidium bromide stained gel of clone cH5 with a small neochromosome that retained CEN9 (center lane; flanking lanes are from other Ura+ clones). The cells retained unrearranged chromosomes II and IX. (B) ArrayCGH confirms that the relevant part of chromosome IX has been duplicated, beginning at the insertion site of ra3; however only the right half of chromosome II is duplicated, ending at the complementary region of the ura insertion. The sequence at ~420 kb could serve as a telomere seed after breakage of the dicentric chromosome. (C) The most parsimonious organization of the duplicated parts of chromosomes II and IX produce a neochromosome that is consistent with the size estimated from the CHEF gel. (PDF) [file pgen.1010850.s005.pdf]

# S6\_Fig: Dicentric → BBF

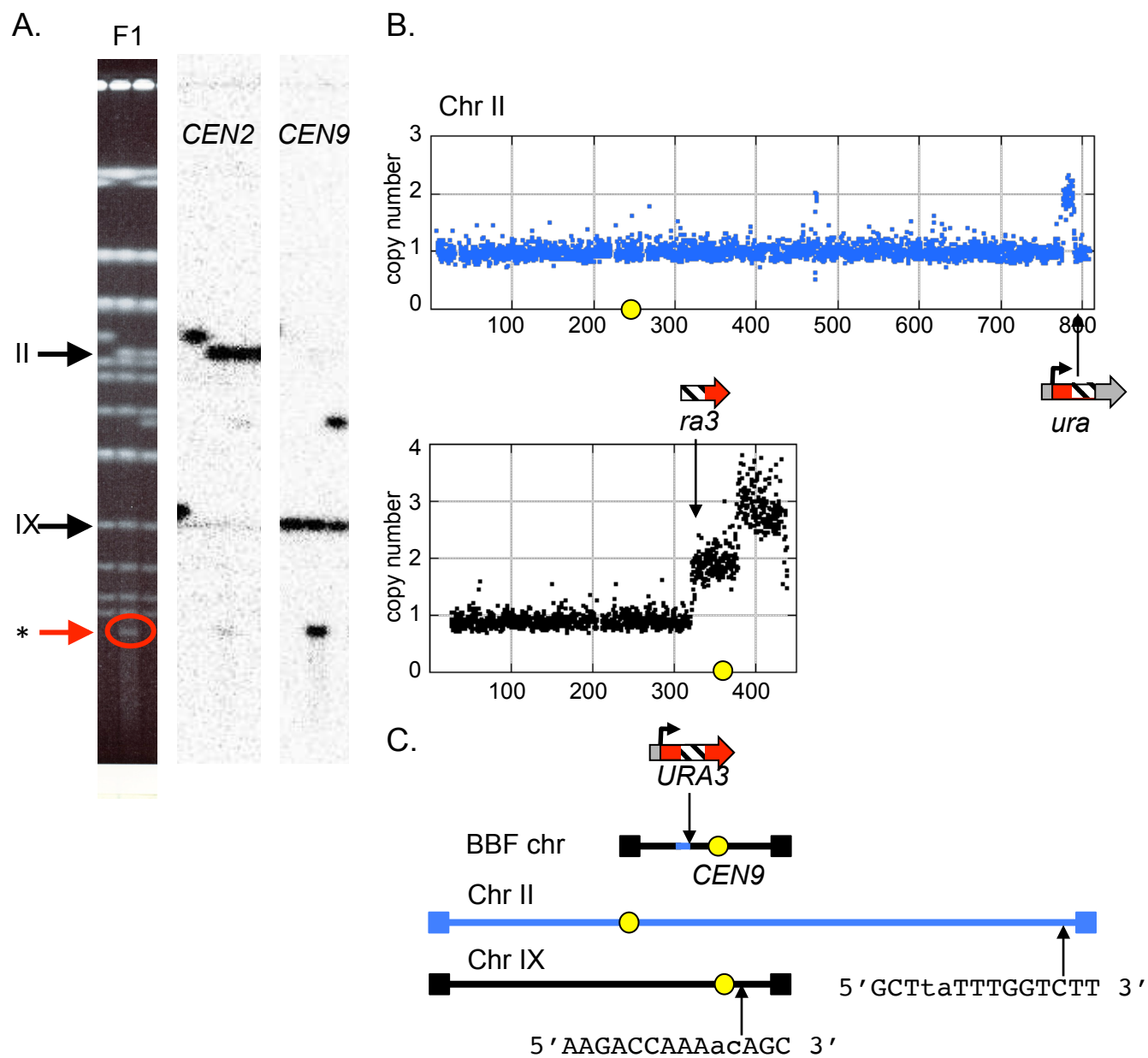

Supplement: S6 Fig — (A) Ethidium bromide stained gel of clone cH5 with a very small neochromosome that hybridizes to CEN9 and the 5’SUL1 sequences from chromosome II. (B) ArrayCGH reveals that only a tiny fragment of chromosome II is retained on this neochromosome and that the left telomere has been replaced by a second copy of chromosome IX right. (C) The most parsimonious organization of the duplicated parts of chromosomes II and IX produce a neochromosome that is consistent with the size estimated from the CHEF gel. Sequences of homology where the second copy of the right telomeric fragment of chromosome IX could have been added are shown in their relative positions on the two native chromosomes. (PDF) [file pgen.1010850.s006.pdf]

S7\_Fig

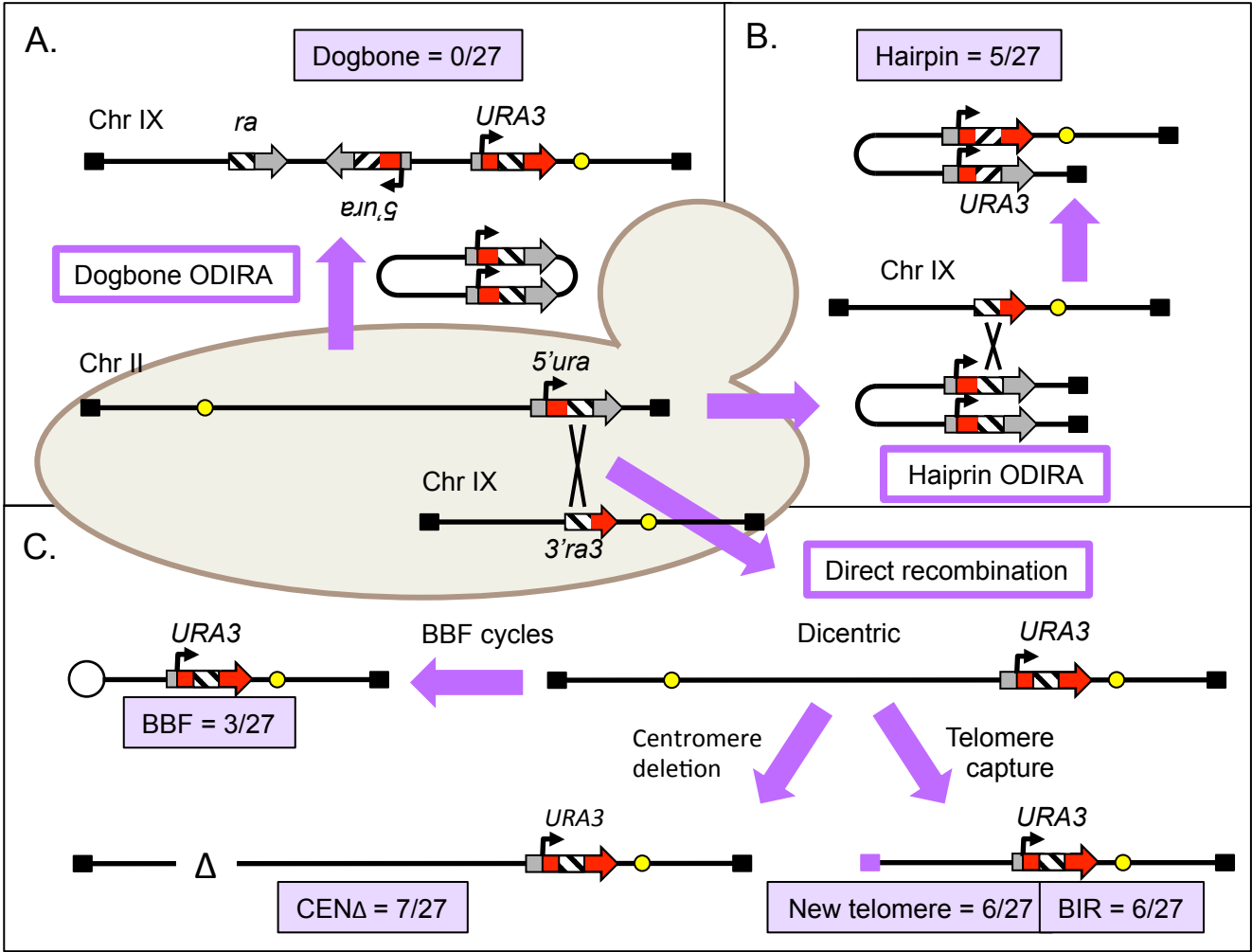

Supplement: S7 Fig — (A) No integration of circular inverted intermediates was observed. (B) Five instances of recombination of an inverted linear with chromosome IX were obtained. (C) The remaining 22 events were produced by direct recombination between chromosome II and IX with subsequent loss of a centromere, breakage and addition of a telomere, or secondary recombination presumably as a result of breakage during mitosis through BBF cycles. (PDF) [file pgen.1010850.s007.pdf]

S9\_Fig

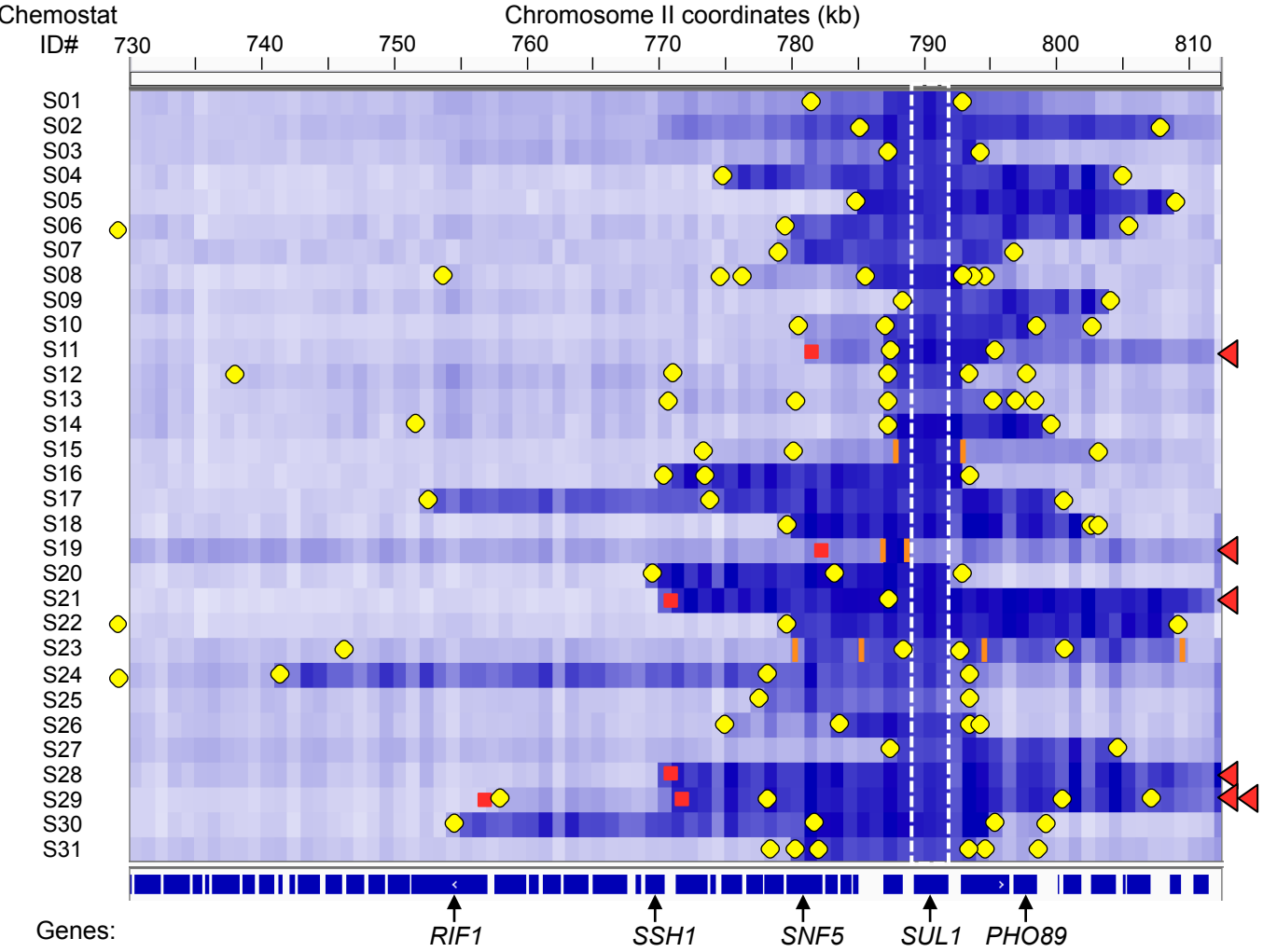

Supplement: S9 Fig — 31 chemostat populations (S01-S31; ~250 generations) were subjected to 150 bp paired end Illumina sequencing. The read depth is shown as a heat map with higher copy numbers in darker shades of blue. The coordinates (in kb) are shown across the top X axis and the positions of ORFS (navy boxes) and several identified genes are shown across the bottom X axis. The position of SUL1 is marked by the white dotted lines. The split reads that mark various types of amplification junctions are indicated by yellow circles (inverted junctions), orange bars (direct repeat junctions), and red squares (junction with an existing telomere—terminal translocations, indicated by the red triangles). Three of the inverted junctions (S06, S22 and S24) occurred in sequences to the left of 730 kb. (PDF) [file pgen.1010850.s009.pdf]

S10\_Fig

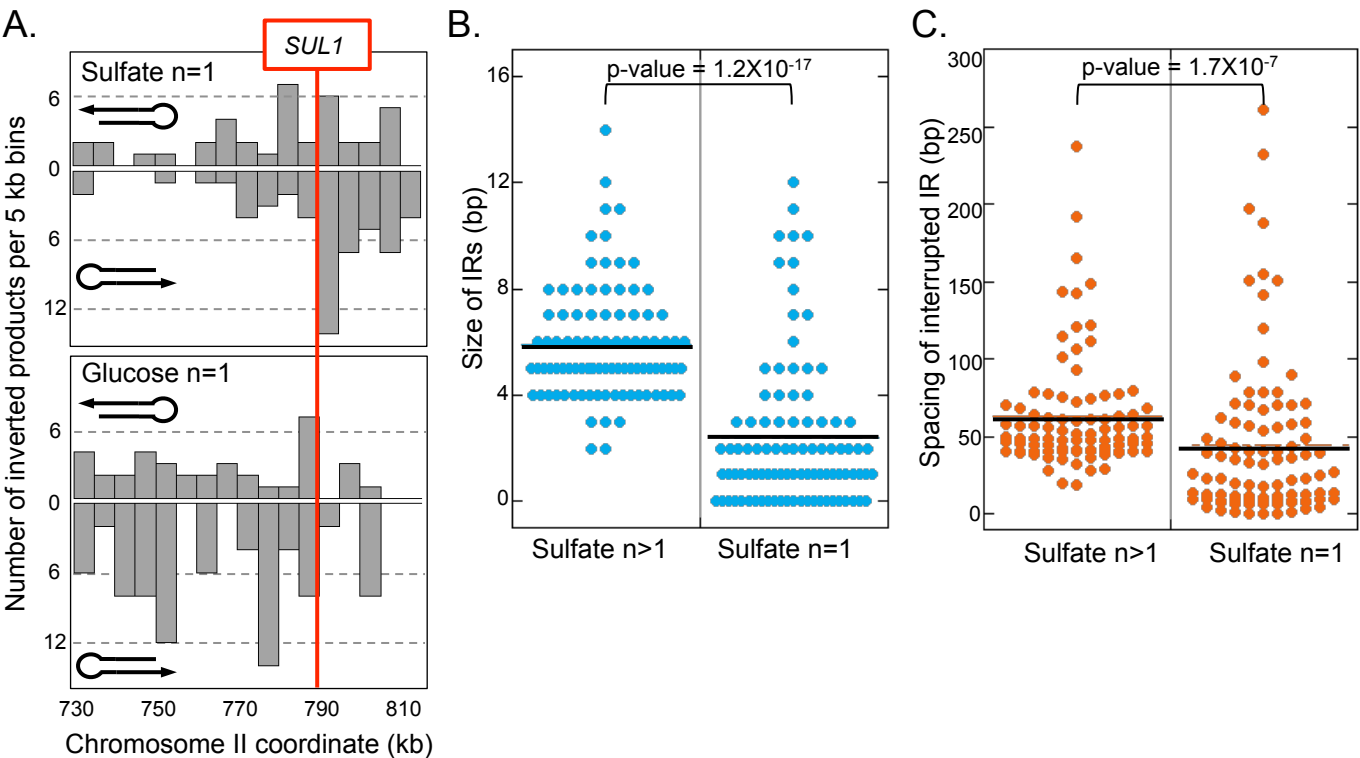

Supplement: S10 Fig — (A) The distribution of inverted Cen- and Tel-junctions recovered from 31 sulfate-limited chemostats (top) and 32 glucose-limited chemostats (bottom) that were represented by single PCR fragment sequences (n = 1). No specific orientation with respect to SUL1 was observed (compare to Fig 5D for split reads with support from two or more PCR fragments). Of note, there were no inverted junctions in the glucose-limited chemostat populations with support from two or more PCR fragments. (B) The size of the inverted repeats from the sulfate-limited chemostats with support from two or more PCR fragments (left) relative to those with support from one PCR fragment (right) are distinctly different. (C) The spacing between the inverted repeats from the sulfate-limited chemostats with support from two or more PCR fragments (left) relative to those with support from one PCR fragment (right) are distinctly different. These results provided the read-depth cut-off for distinguishing PCR artifacts from bone-fide in vivo inverted junctions. Data for all inverted junctions (n>1) in the sulfate-limited chemostats are in S6 Table. Data for all inverted junctions (n = 1) in the sulfate- and glucose-limited chemostats are in S7 and S8 Tables, respectively. (PDF) [file pgen.1010850.s010.pdf]

S11\_Fig

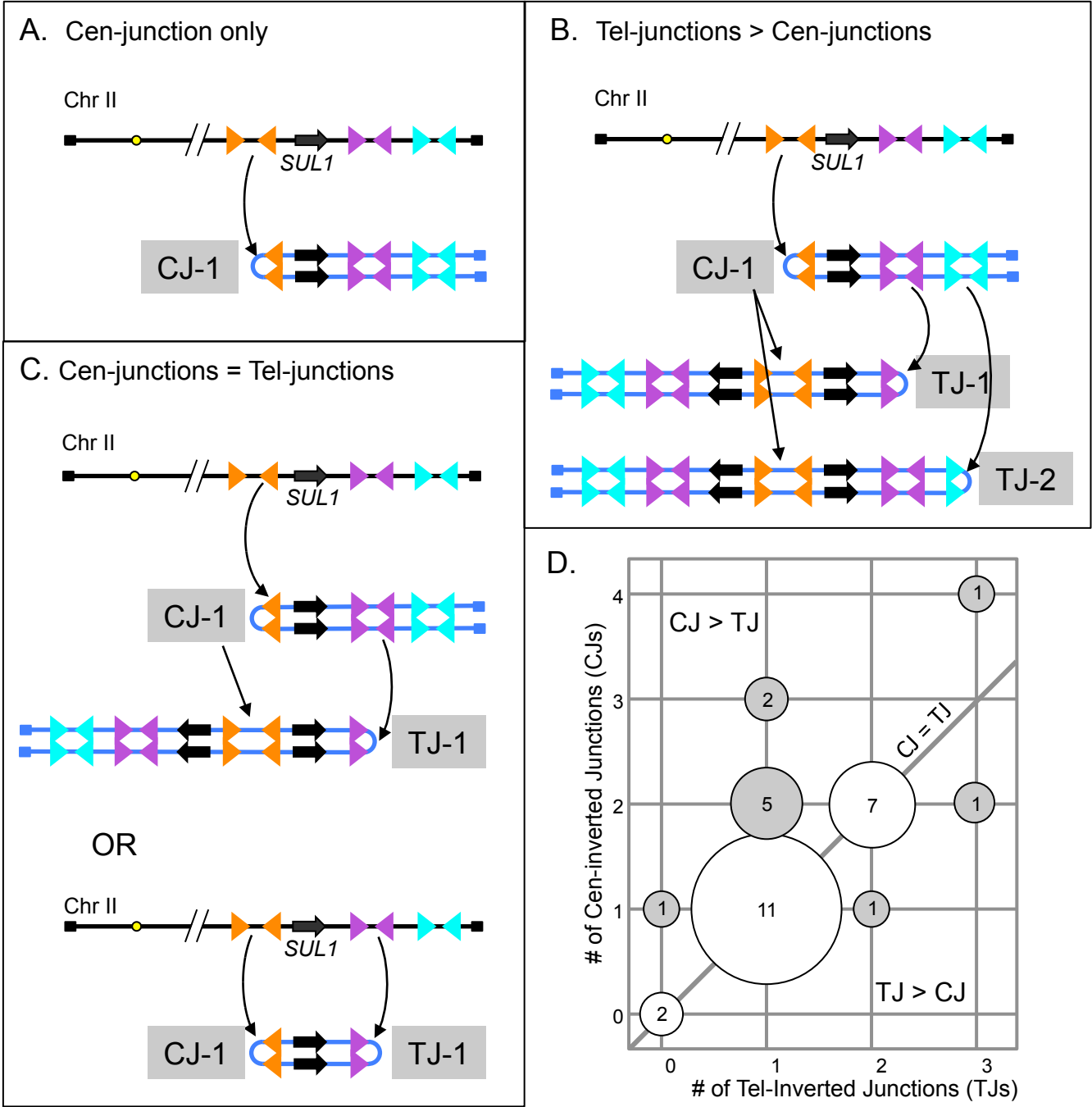

Supplement: S11 Fig — If inverted amplicons of the SUL1 region arise through hairpin intermediates then in each population the number of Cen- and Tel-junctions would not necessarily be equal. (A and B) Mechanisms to explain unequal numbers of Cen- and Tel-junctions. Colored triangles indicate different inverted repeats along the chromosome. (C) Mechanisms to explain an equivalence between Cen- and Tel- junctions. (D) Among the 31 sulfate-limited chemostats, two had no inverted amplicons of the SUL1 gene (one was a tandem duplication and the other was an amplification of the SUL1 promoter region). The remaining 29 produced the total number of 92 inverted junctions. Eighteen cultures had matched numbers of Cen- and Tel-junctions. The remaining eleven had unbalanced numbers of Cen- and Tel-junctions. (PDF) [file pgen.1010850.s011.pdf]
