# Supplementary material for: Template switching between the leading and lagging strands at replication forks generates inverted copy number variants through hairpin-capped extrachromosomal DNA
Source: PLoS Genet. 2024 Jan 4;20(1):e1010850. doi: 10.1371/journal.pgen.1010850 (PMC10766183; doi:10.1371/journal.pgen.1010850)
Supplement: S8 Fig — (A) Repair of a double stranded break could expose an inverted repeat in the 3’overhang that could be repaired to create the hairpin linear and its replicated isochromosomal fragment. If this double stranded break repair mechanism is responsible for the inverted Ura+ clones then CRISPR/Cas9 directed cutting on the Cen-proximal side of the SUL1 region should lead to an increase in Ura+ clones overall and an increase frequency of inverted outcomes. (B) Resection of the 5’end of a double strand breaks introduced distal to SUL1 would expose the ura homology in single stranded form and stimulate recombination with the ra3 sequences on chromosome IX. CRISPR/Cas9 cleavage on the Tel-proximal side of SUL1 should increase the overall frequency of Ura+ clones that occur through recombination events between the two chromosomes. (PDF) [file pgen.1010850.s008.pdf]

S8_Fig

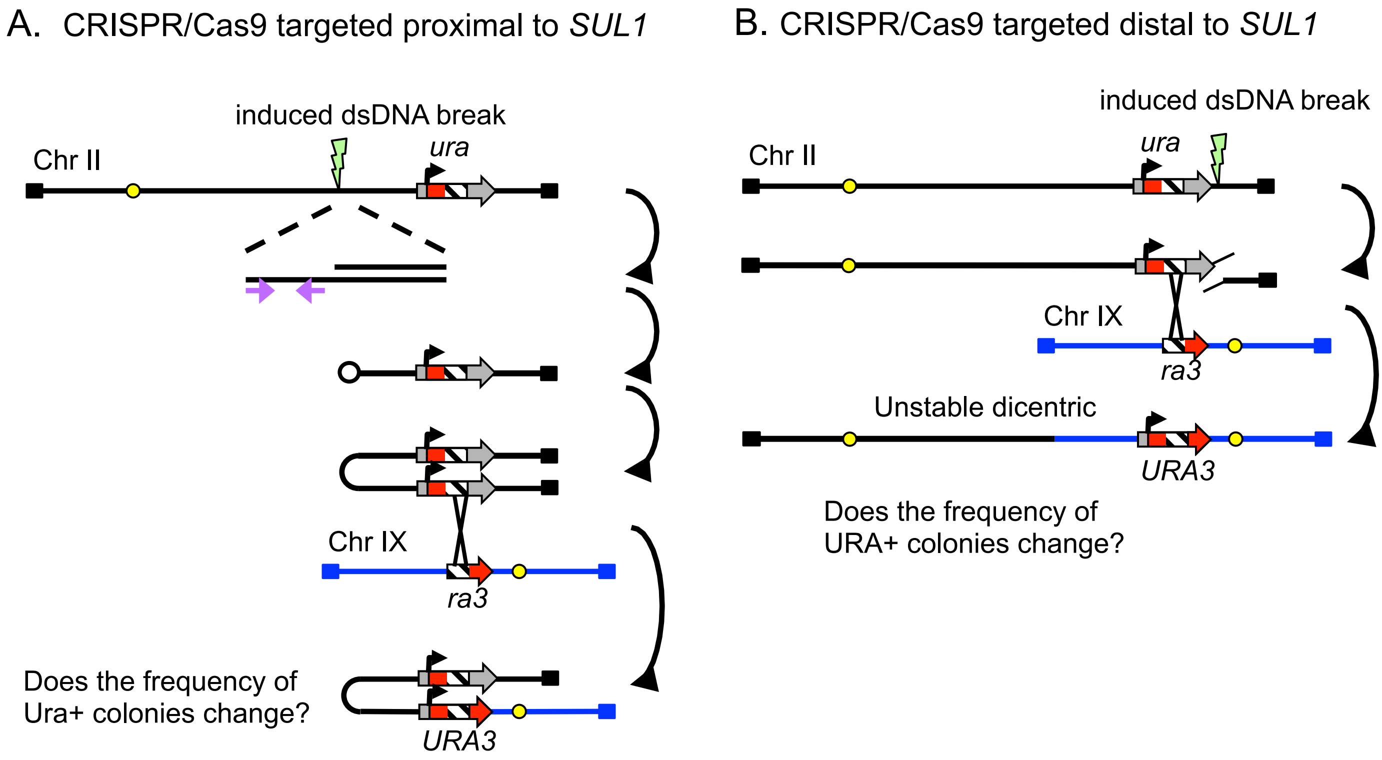
A. CRISPR/Cas9 targeted proximal to SUL1
induced dsDNA break
Chr II
ura
Chr IX
ra3
Does the frequency of
Ura+ colonies change?
URA3
B. CRISPR/Cas9 targeted distal to SUL1
induced dsDNA break
ura
Chr II
Chr IX
ra3
Unstable dicentric
URA3
Does the frequency of
URA+ colonies change?
