# Supplementary material for: Template switching between the leading and lagging strands at replication forks generates inverted copy number variants through hairpin-capped extrachromosomal DNA
Source: PLoS Genet. 2024 Jan 4;20(1):e1010850. doi: 10.1371/journal.pgen.1010850 (PMC10766183; doi:10.1371/journal.pgen.1010850)
Supplement: Attachment — Submitted filename: Response to reviewers PLoS Gen Martin et al.docx [file pgen.1010850.s021.docx]

**Comments to the Authors:
Please note here if the review is uploaded as an attachment.**

We would like to thank David Gresham and the two anonymous reviewers for their thorough and thoughtful reviews. The manuscript is improved as a result of their efforts. Below are our responses to each of their individual comments.

Reviewer #1: Martin et al investigate the mechanism underlying a specific class of copy number variation (CNV). Previously, this group has proposed a DNA replication-based mechanism, origin dependent inverted repeat amplification (ODIRA), to explain the occurrence of CNVs that are distinguished by a triplication with the internal repeat segment present in the inverse orientation and the flanking regions of the CNV containing short inverted repeat sequences. The proposed mechanism has been used to explain amplification at the SUL1 locus in yeast lineages that arise during the course of adaptation to sulfur limited chemostats. It has also been suggested that this mechanism underlies some CNVs at the GAP1 locus in yeast lineages that arise during glutamine limited chemostat selections. A similar class of CNV has been reported in the human genome making an understanding of this mechanism of CNV formation of potentially broad interest.

In this study, the authors engineered a yeast strain with the goal of distinguishing alternative variants of the ODIRA mechanisms and distinguishing them from recombination based mechanisms. A split URA3 gene was created with half the gene on chromosome IX and half the gene on chromosome II. The authors selected for ura+ clones that generated a functional URA3 and resolved the chromosomal structures using a combination of CHEF gels, southern blotting, and array comparative hybridization. Based on these analyses the authors propose that ODIRA occurs through an extrachromosomal linear intermediate that they term a hairpin rather than a circular intermediate as previously proposed in their model. To further test the role of DNA replication the authors treat the cells with hydroxyurea and introduce DNA breaks. Finally, the authors analyze additional populations of yeast cells selected in sulfur limited chemostats to characterize putative ODIRA events at the SUL1 locus.

This is an interesting study that contributes to our understanding of the proposed mechanism of CNV formation. In general, the experiments are well-performed and analyzed. Thank you. Prior to publication, the authors should address the following:

-I had a hard time understanding Figure 1, which should be improved to clearly explain the difference between the two proposed intermediates. I suggest

1) labeling the telomeres Done.

2) clearly distinguishing between ssDNA and ssDNA with a consistent color or line type. Thank you, we have modified all of the figures in which both single stranded and double stranded DNAs are depicted. In Figure 1 the molecules in the gray rounded rectangles now have thin lines for the two strands of the double helix. Lines representing double stranded DNA are thicker. In the dogbone, before it is replicated to create a dimeric inverted circle, the main body is double stranded (thick line) with single stranded loops at the ends (thin lines). The same is true for the hairpin figure. The blue color of the lines is indicating the fate of the ODIRA molecule—the original strands joined by template switches. We added clarifying language to the legend of Figure 1.

3) when the “hairpin” molecule is dsDNA it is no longer a hairpin and so should be drawn as linear molecule to make this clear. We have changed the way we show the doubly inverted linear (Figure 1B.5). After replication the two halves of the doubly inverted construct are folded back on one another to emphasize the identical DNA sequence that then aligns with the same sequence in the unrearranged (black) chromosome. We have also described that recombination with either of the two inner copies generates the inverted triplication in the chromosome while recombination with either of the outer two copies just swaps telomeres (page 6).

4) use a different symbol for centromeres (e.g. circles) and origins (e.g. a square) rather than just a different color. Done. Centromeres are now stars.

-Similarly, I had a hard time understanding Figure 2. I suggest using a consistent color/symbol system (as with Figure 1). Done—the color system is now defined in the legend to Figure 2. It would be helpful to clearly indicate what the final products are (e.g. with some shading or an outline) versus the intermediates to understand the rationale for the expected CHEF gel and southern results. Good suggestion. The final products are included in dotted rectangles in Figure 2.

-I think that Figure 1 implies that the final product from a circular or linear intermediate is identical. Is that correct? If so, it provides an additional justification for using the split ura system and this could be made clearer in the text. Yes, for *SUL1* amplification in chemostats the two models produce the same outcome. However in the split-URA system we can capture the first hairpin product, so the Ura+ product we recover is not identical to the outcome of the dogbone model. Because we didn’t recover the expected inverted triplication, it suggested to us that there was an intermediate step in the ODIRA mechanism. Thank you for making this suggestion to be more explicit in the text. We have included this point in the new Discussion section (pg 31).

-As the hairpin model is drawn in figure 1, there are four copies on the linear intermediate. I would think this could result in either 4, 3, 2, or 1 copies when it recombines with the chromosome depending where the crossover occurs. Is this correct and if so are these variable number copies observed in sulfur limited selections? No, only 1 or 3 copies can be created on chromosome II by recombination with the doubly inverted linear intermediate. Using either of the terminal copies for recombination doesn’t change the number of copies on chromosome II—the telomeres just get swapped. If either of the two central copies is used, then the product on chromosome II has 3 copies. That is identical to what one would see if the inverted dimeric circle had integrated into the chromosome and is what we commonly see in the sulfate limited chemostat cultures—odd numbers of copies but never even numbers. We have changed the drawing of the doubly inverted linear recombining with chromosome II in Figure 1B.5 to make this point more clearly.

-In the summary and abstract the authors make a connection to this class of variation in humans, but do not address the connection in the results or discussion. It would be worth addressing in the discussion how relevant the results are to understanding the mechanism in humans and/or its distinction from FoSTeS. Is it possible to undertake an analysis of inverted repeat frequency in the human genome and/or look at short read data for evidence of the breakpoints occurring at IR sequences? These questions are really interesting and important but a significant discussion of them is too much for this paper. We have included a short statement regarding human inverted CNVs in the new Discussion section (pages 32-33). We have submitted a second manuscript to PLoS Genetics (and currently available on biorxiv, <https://www.biorxiv.org/content/10.1101/2023.09.19.558550v1>) with a more in-depth analysis of ODIRA-generated CNVs in humans that we hope will accompany this yeast CNV paper.

- The reference to the human study in which a 2:1 SNP ratio was observed suggests that a useful experiment would be to perform sulfur limited chemostat selections using a hybrid strain (e.g. BYxRM). Have the authors tried this? Would the outcome of this approach be informative about the underlying mechanism? We did this experiment with a diploid that had an internal small deletion distal to the *SUL1* locus on chromosome II that we could use to distinguish the two chromosomal copies but found that this particular diploid amplified *SUL1* as short linear fragments with a new telomere added to the proximal junction (Hoerr RE et al., 2023, PMID 36702776). This result necessitated the creation of the split–Ura3 strain. We still do not know if this difference is a peculiarity of the lab strain we used or a real difference between diploids and haploids. The split-ura system should help us investigate this question in the future.

- Given that chemostat selected populations are heterogeneous the presence of multiple CNV clones can confound the resolution of breakpoints. The authors appear to be successful in doing this. Populations adapted to glucose are used as a control, but I would think a comparison to sequenced clones in which only one breakpoint is identified would be a better comparison as it includes a positive and negative control. The authors had a discussion on this point and came to differing opinions about what was being asked. We previously sequenced 7 clones with 75 bp reads. Because of the shorter read lengths, it is more difficult to identify inverted junctions, however, in all seven cases there were just two inverted junctions per clone and in the 81 kb region of chromosome II there were no other split reads with more than a single read. There were so few that we wonder whether we had enough power to confidently make a read number cut-off for the negative control using just the data from the 7 clones. We would need to sequence 30 clones with 150 bp reads to get the same level of confidence for our cut-off. We have not done extensive sequencing of clones.

-The lack of inverted sequences in the glucose-limited chemostat is surprising. Is it not possible that amplifications that include HXT6/7 can also be generated through ODIRA? We only looked for split reads in the 81 kb of chromosome II. In response to this question, we went back and specifically looked for split reads in the *HXT6/7* region of chromosome IV. In the 32 glucose chemostats, only two had evidence for *HXT6/7* amplification (there is a fitness cost associated with this amplification event) and there were no inverted split reads of n>1 mapping in this region. Because the *HXT6/7* genes are in direct orientation and are highly homologous (99%), amplification is achieved by unequal sister chromatid recombination that expands the region as a series of direct tandem repeats. The junctions would not be inverted and direct repeat junctions would lie in the regions of perfect homology and thus be undetectable by 150 bp sequencing reads.

-The interpretation for the “waterfall” signal should be better explained in the results, perhaps with a cartoon to explain how this signal is consistent with an inverted junction. It is not intuitive to me. Figure S2 is a cartoon explaining the “waterfall” pattern on aCGH. We were wondering whether David overlooked this figure or if he didn’t think it was clear. We have added another panel to the figure showing the DNA after denaturation in preparation for *in vitro* labeling with cy dyes. Those 20 kb fragments that span a portion of the inverted junction snap back and are not accessible to the primers and polymerase that are used to incorporate the cy dyes and thus the duplex regions in those snap-back molecules cannot contribute to an increase in signal on aCGH. Because these large fragments have different ends, there will be different lengths of snap-back DNA with the actual junction being underrepresented to the greatest degree.

- If addition of HU inhibits the ODIRA mechanism one would expect that the overall rate of ura+ clones is reduced. The authors state that the proportion of inverted clones is reduced, but is the overall rate reduced? We did not measure rates, just frequencies of Ura+ clones. We found that the overall frequency of Ura+ clones did increase upon growth in HU (3.5-fold in 50 mM and 5-fold in 200 mM) but only the Ura+ clones that were the product of direct recombination showed this increase. The number of inverted clones did not increase. We agree with your concern (and the concern of the other two reviewers) that the reduction might be explained by an increase in direct recombination products rather than by interference with template switching at the HU challenged forks. However, the fact that we don’t see an elevation in inverted Ura+ clones suggests to us that any DNA breaks occurring centromere-proximal to the *sul1::ura* locus are not the initiating event in inverted Ura+ events while they are very likely the cause of increased direct recombinant types that are produced by DNA breaks distal to the *sul1::ura* locus. We now discuss this point in the manuscript and have moved this section to precede the CRISPR section (pages 20-22). Thank you for pointing out your concerns that necessitated our reevaluating these data.

- On page 26 it is unclear if the distribution of IRs is for chr II or the whole genome as the authors refer to “the genome”. We deleted “in the genome”. We only looked at this 81kb region but we have no reason to suspect that it isn’t uniform across the genome.

-The role of a circular and linear intermediate could be tested by synthesizing this element and testing whether it generates the expected product. As written it is unclear whether this has been tried for the dogbone model (see page 26) and/or if it was attempted in this study for either intermediate. An artificial dogbone was created *in vitro*, introduced into yeast and results for its persistence and recombination with its target site in the genome were characterized in Brewer et al., 2015 (PMID 25749076). One of the transformants had a secondary rearrangement in one of the palindromic junctions. It is the same reference in which we found a secondary rearrangement of a chemostat derived amplicon.

- The authors’ study is focused on SUL1, but they neglect to point out that there is evidence that ODIRA underlies CNVs at the GAP1 and DUR3 locus (see Lauer et al., PLoS Biology 2018). Mentioning this could increase the relevance of this study. Absolutely, thanks for pointing out the omission, we should have mentioned this work. We have included a reference to the Lauer study on page 27. However, we prefer to say that the results of Lauer are consistent with ODIRA and not direct evidence for ODIRA.

- Some of the properties of ODIRA breakpoints at the SUL1 locus that are described have been reported in Lauer et al. 2018 - e.g. IR length and spacing (see figure 4). It would be interesting to compare the properties of SUL1 ODIRA breakpoints to these data to determine how generalizable they are. You are correct and they are very similar. We have added this comparison on page 27.

Reviewer #2: The manuscript by Martin, Espinoza et al extends the work of the Brewer / Raghuraman group in the characterization of the ODIRA mechanism these authors proposed from their earlier analyses of amplification events spanning the SUL1 locus in S. cerevisiae. ODIRA is a somewhat unique pathway in that it is associated with DNA replication and leads to a hallmark triplication with characteristic orientation and short inverted repeats. The SUL1 locus contains the right combination of ingredients for this class of events to occur and then expand clonally and be detected among S. cerevisiae cells evolved under sulfate limiting conditions. Interestingly, the signature rearrangement configuration found in SUL1 triplications resembles certain (even more) complex disease-associate triplications in the human genome, suggesting possible universal consequences of ODIRA to genome instability across species. The Brewer / Raghuraman group has steadily refined their characterization of ODIRA, and in this manuscript they continue to advance their work in two valuable ways.

First, they set up a clever system for interrogating the structural nature of the proposed aberrant replication DNA unit that serves as the key intermediate in ODIRA triplications. The experimental system described here is particularly significant because it offers a substrate for triplications to re-integrate on Chr IX, outside of the SUL1 locus in Chr II. This movement provides an important example for how ODIRA can lead to structural variation even at distant recipient loci that lack the requisite replication origin flanked by pairs of short inverted repeats. Notably, the results of these experiments prompted the authors to revise their own original model in favor of a hairpin intermediate, replacing the circular ODIRA intermediate they had proposed earlier. Their embracement of a new data-driven revision of their original model was quite refreshing to read. Thank you.

The second advancement in this manuscript is a thorough analysis of ODIRA junctions among cultures evolved in sulfate-limited chemostats. By analyzing bulk WGS reads from cultures (rather than purified clones), they were able to increase the number of junctions scored, and then used that information to infer mechanisms and propose novel ODIRA details.

Overall, this clearly written manuscript presents rigorously conducted work that further advances our understanding of the ODIRA mechanism. The manuscript does open many new questions, and leaves most unanswered. Several aspects of ODIRA are still uncharacterized, for example, the identity of the enzymes that must carry-out the various steps, and the direct isolation and identification of the hairpin DNA intermediate. Nevertheless, this work provides valuable new insight into one of the ways in which inappropriate DNA replication can drive the formation of certain classes of chromosomal alterations, and structural genomic variation in other broader contexts.

Below are major and minor suggestions that authors and editors should consider to improve the manuscript, ordered as they appeared in the manuscript text:

Major:
-The final paragraphs of the Introduction read very much like they belong in the Results section. I suggest keeping the rationale short in the Introduction, and expand on the details of the approach in the Results. We have addressed these comments in the final paragraphs of the introduction.

Line 462, and the whole HU experiment. This is my biggest comment and concern. I disagree with the interpretation of the HU results. An alternative (an in my view more likely) possibility is that replication stress caused by the HU treatment simply increases direct ura/ra3 recombination generating more dicentrics among the Ura+ clones analyzed (similarly to the case of the distal [792kb] CRISPR DSB). In this plausible scenario, the proportion of ODIRA Ura+ clones would be lower with HU exposure than without. Treating cells with gamma-rays or other global recombinogenic agent unrelated to replication stress would lead to the same outcome. The current HU interpretation can only be supported directly if the authors are able to determine and compare the absolute (not relative) number of inverted Ura+ clones with and without HU. We did not measure rates, just frequencies of Ura+ clones. We found that the overall frequency of Ura+ clones did increase upon growth in HU (3.5-fold in 50 mM and 5-fold in 200 mM) but only the Ura+ clones that were the product of direct recombination showed this increase. The number of inverted clones did not increase. We agree with your concern (and the concern of the other two reviewers) that the reduction might be explained by an increase in direct recombination products rather than by interference with template switching at the HU challenged forks. However, the fact that we don’t see an elevation in inverted Ura+ clones suggests to us that any DNA breaks occurring centromere-proximal to the *sul1::ura* locus are not the initiating event in inverted Ura+ events while they are very likely the cause of increased direct recombinant types that are produced by DNA breaks distal to the *sul1::ura* locus. We now discuss this point in the manuscript and have moved this section to precede the CRISPR section (pages 20-22). Thank you for pointing out your concerns that necessitated our reevaluating these data.

-The Conclusions section is limited to the findings of the work, which I guess is appropriate. However, the manuscript is lacking in terms of discussion of the findings in light of the broader genome instability field. I believe the conclusions should be folded within a proper Discussion section. Some of the suggested points for that expanded section include: We have added a discussion section instead of a conclusion section (pages 31-33).

i. It would be useful to discuss the broader implications of ODIRA in other system, in light of the findings obtained in this study. We agree but a significant discussion of ODIRA in other systems (ie. Humans) is too much for this paper. We have included a short statement regarding human inverted CNVs in the new Discussion section (page 32-33). We have also submitted a manuscript on human inverted CNVs to PLoS Genetics (and currently available on biorxiv at <https://www.biorxiv.org/content/10.1101/2023.09.19.558550v1>) that we hope will accompany this manuscript on yeast CNVs.

ii. Another point that could be addressed is something about the duration of the hairpin intermediate, or approaches that might be used to eventually capture it for analysis. How ephemerous is it? Can one ever hope to isolate it? We imagine that the dogbone or hairpin intermediate would persist until the next cell cycle when it undergoes replication from its resident origin. We have little hope of isolating it from either the chemostats or in the split-ura3 strains. We have emphasized their ephemeral existence in the text and the impossibility of capturing them by DNA sequencing (page 24).

iii. Finally, consider points about potential roles of sequence-specific endonucleases during the early steps in ODIRA. The step immediately following the template switch (Fig. 1A2) creates structures that might be recognized and cleaved by some of these enzymes. Would absence of those activities allow more of the strand switches to remain intact and thus progress toward mature hairpin intermediate formation? These could be in some way suppressors of ODIRA. Suggested looking at some of the work reviewed in PMID: 34624742. Good idea. The fact that we find so few junctions in the population sequencing suggests that the error of strand switching between the leading and lagging strands must be rare. However, it is also possible that the replication error is actually more frequent but that many of the dogbone/haipins are processed by cleavage and repair enzymes before they can undergo replication in the subsequent cell cycle. We have included a short discussion of this possibility in the discussion (page 32) and considered both mutations that suppress ODIRA events and those that might stimulate them. It was not possible to get enough replicates of mutant strains to ask these questions using chemostats, but now we have the split-ura3 system, and have started creating some of these mutants.

Minor:
Line 24: “Inherited and de novo copy number variation…” I think this sentence should be revised as some de novo mutations are actually inherited (mutations formed de novo in a parent during gametogenesis). We replaced “de novo” with “germ-line de novo” to distinguish them from somatic mutation or preexisting CNVs inherited from a heterozygote.

Line 74: “and template switching during replication (FoSTeS and MMBIR)”. Note that the term “template switching” used here and other points in the text, has also been used extensively in association with BIR, particularly work from L. Symington’s lab (starting in 2007 with Smith et al PMID: 17410126). Given that this manuscript addresses genome rearrangements in yeast, I think it is important to specify precisely of what is meant by “template switching” here (eg. switching from leading strand template to lagging strand template). Template switching is also used extensively in papers that promote FoSTeS (Fork stalling and template switching) as the mechanism for complex human CNVs. And since this mechanism is a replication based one, we didn’t think that it needed further clarification. We define what we are calling template switching in the abstract, summary, introduction and the opening sentence to the discussion. And it is illustrated in detail in Figure 1. We hope that these clarifications sufficiently address the reviewer’s concern.

Line 75: Some of reviews cited here (7-12) are already getting a bit old, and therefore not include important advancements in the field. For example, whenever discussing mechanisms that involve microhomologies, it is essential to include mention of relatively-recent advancements in MMEJ mediated by Pol Theta (even though it is absent in S. cerevisae). That specific topic can be referenced for example from PMID: 32302896. We have added this reference (page 4).

Line 163: “Together these results suggest that double-stranded DNA breaks are not…”. I do not agree with this statement. It needs to be revised. Maybe DSBs are not needed to generate the extrachromosomal intermediate (dogbone or hairpin; Fig. 1A4 and 1B5), but a DSB in that intermediate or in the chromosomal integration locus would be needed to trigger the final step in ODIRA (HR-mediated re-integration of that intermediate into the chromosome). You are correct. Thanks for catching that oversimplification. We have made the appropriate change on page 8.

Line 197: I think it would be important to give the ra3 and ura integrations specific allele names that can be included in the genotype of strain S2-1. As presented, the strain’s genotype give no indication of the presence of these repeat substrates. One possibility it to designate them as insertion downstream of the nearest gene (eg. YFG1::ra3 and YFG2::ura)(YFG, Your Favorite Gene), or something else to indicate they are in the strain. We have indicated the locations of the two insertions with coordinates and nearest genes in the methods section (page 10-11).

Also related, why are ra3 and ura sometimes underlined in the manuscript? It was unclear to me what was the meaning to the underlining. We have removed the underlines.

Line 341: “Because the left arm of chromosome IX has no centromere…” Sentence needs revision. By definition, no chromosome arm can have a centromere, otherwise it would not be an arm. Correct. Thanks. We have changed this sentence to read “The acentric fragment that contains the left telomere would be lost . . .”

Line 403: The terms “circular” and “dogbone” are sometimes used interchangeably. It would be best to use a single term throughout, in which case, it would be “dogbone” because it is more unique and catchy – readers will know exactly what it is about. Dogbone refers to the fleeting intermediate that is expelled from the chromosome after an adjacent fork dislodges it. After that replication converts it to an inverted dimeric circle. We have scanned the manuscript to be certain that we are being consistent. Likewise, hairpin is the transient intermediate. We now refer to its replicated form as inverted linear.

Reviewer #3: The focus of the manuscript from Dr. Brewer and her group is the new mechanism called ODIRA generating segmental inverted triplications similar to those frequently associated with human developmental and cancerous phenotypes. The idea of the elegant new mechanism proposed by the authors is that following replication pausing and fork regression, template switching occurs at inverted repeats followed by ligation of the leading and lagging strands. The authors propose that this event can generate an extrachromosomal intermediate that can later integrate into the chromosome generating segmental inverted triplications. The authors proposed two different variations of their model, built an experimental system in yeast to test these two variations of their ODIRA model, and successfully tested their predictions using their experimental system. The results of their test support the existence of one of their proposed mechanisms, hairpin ODIRA, but not another one, dogbone ODIRA. Based on their results, the authors also propose that replication stalls (and not DSBs) serve as the main inducers of ODIRA-events. In addition, the authors report the optimal parameters of short inverted DNA repeats mediating ODIRA. Together, the authors conclude that their data support a two-step version of ODIRA model, where sequential template switching events at short inverted repeats between leading and lagging strands at replication forks generate inverted interstitial triplications. The results presented in this elegant and detailed manuscript will be of great interest for the diverse readership of PLoS Genetics including researchers studying the mechanisms of chromosomal rearrangements including those leading to congenital diseases, cancer and to neurological syndromes. Thank you.

Specific comments:

1. How did the authors decide on the position for the insertion of the “a3 piece”? I mean is there any reason for inserting it into the Chr IX and at this specific location at this chromosome? We wanted to target integration into a small-ish chromosome so that we could measure increases in chromosome size easily and accurately on CHEF gels (and chromosome IX is well separated from the larger chromosomes, giving us lots of room on the CHEF gel to see altered sizes). We chose the orientation for the *ra3* sequence to point towards the centromere to reduce the chance of recovering direct recombination products. In this orientation direct recombination produces a dicentric that would require secondary rearrangements to produce a stable Ura+ clone. Finally, we looked for a non-essential site between genes, as close as possible to the centromere, for the integration site. We did not include this reasoning in the methods section. We could if you think it is necessary.

2. P. 21, line 462-464. Following the exposure to HU, the authors observed the decrease of the fraction of inverted Ura+ clones among all selected Ura+ events. This led them to conclude that ODIRA is decreased in the presence of HU. What if HU exposure led to the increase of the competing events leading to Ura+ instead? For example, could the result be explained by HU stimulating chromosome breakage promoting direct recombination between Chromosomes II and IX producing Ura+? We did not measure rates, just frequencies of Ura+ clones. We found that the overall frequency of Ura+ clones did increase upon growth in HU (3.5-fold in 50 mM and 5-fold in 200 mM) but only the Ura+ clones that were the product of direct recombination showed this increase. The number of inverted clones did not increase. We agree with your concern (and the concern of the other two reviewers) that the reduction might be explained by an increase in direct recombination products rather than by interference with template switching at the HU challenged forks. However, the fact that we don’t see an elevation in inverted Ura+ clones suggests to us that any DNA breaks occurring centromere-proximal to the *sul1::ura* locus are not the initiating event in inverted Ura+ events while they are very likely the cause of increased direct recombinant types that are produced by DNA breaks distal to the *sul1::ura* locus. We now discuss this point in the manuscript and have moved this section to precede the CRISPR section (pages 20-22). Thank you for pointing out your concerns that necessitated our reevaluating these data.

3. Fig. 1B and the text of the paper: It is unclear how the extrachromosomal piece (the very bottom, right) ended up with two pairs of the purple inverted repeats. If one follows the cross-over, beginning with the centromere of chromosome II and moving right, it joins with the third repeat in the doubly inverted linear. The end of chromosome II then contains the end of the doubly inverted linear and picks up two sets of the purple repeats. We have amended that part of the figure to make the recombination outcome a little easier to understand.

4. Could the authors explain in detail the formation of the outcome J2 (Figure 3A). The neochromosome in this outcome appears very large for the product of dicentric, and it is not clear how it could be formed via the proposed ODIRA mechanism. At least some information on the size of the product and possible mechanism would help. J2 is not dicentric but is the product of a recombination event between the inverted linear produced by ODIRA and chromosome IX (through the ra homology), acquiring *CEN9* and the whole right arm of chromosome IX. The aCGH data for J2 is shown in Figure S3. The region from chromosome II that is included in the initial hairpin linear is quite large (nearly 500 kb that would be present twice). Once that inverted linear recombines with chromosome IX, ~30 kb of one of the chromosome II right telomeres is replaced by the centromere and right arm of chromosome IX (~100 kb). The new chromosome is approximately 1000 kb. While many of the neochromosomes are small, we do recover larger ones such as J2 and G3A (Figure S3). We mention these two clones on page 20.

5. Fig. 3C: it would help to indicate the specific location/chromosome region hybridizing to the probe “x”. The primers used to create the three probes are included in a new Table S2.

6. The data presented in the Figure 5B are not clear. More explanation is needed to this panel in general. Also, some specific details need to be explained. For example, what is depicted as thin stripes of different colors? This image is generated in the program IGV and the colored bars indicate a single base pair difference from the reference sequence. Most of the reads are perfect matches but some have one or more sequencing errors that do not interfere with their ability to be mapped to the genome. We included this information in the figure legend on page 38.

7. Fig. 5D: not clear what is the meaning of asterisks. The asterisk in 5B highlights the specific read that is illustrated in 5A and its position to the right of *SUL1* in 5D. We have added this explanation to the figure legend (Page 39). Thanks for pointing out this omission.

8. Fig. 6D: the legend mentions a striped arrow, but it is unclear which one. I did not find it. The striped arrows are mentioned in Figure 6F, not 6D. They refer to replication forks that are moving in a direction that could not produce the centromere-proximal and telomere-proximal junctions for *SUL1* amplification (Page 29).

9. Fig. S3 and the text: the authors compare the left and the right boundaries of the amplified region and indicate that the right is more abrupt than the left one. It is hard to tell. How rigorous this comparison was? It seems like there is a lot of variation. The right junction is created by recombination within the ra sequences at the two locations (Chr II and Chr IX). That is the only place where a junction could occur and create a Ura+ gene. There is a clean jump in copy number at this location, whereas, the left margin is created by ODIRA and produces the “waterfall” pattern on aCGH. These individual clones have centromere-proximal junction sites that are widely spaced in the ~500 kb centromere proximal to *SUL1* (Page 19).

10. Figure 7C, the last, right, column: is it right that the loop is formed in the blue (newly synthesized) strand? My impression is that the black strand will need to loop out. We are not completely certain what is being asked. After fork regression, the black strand doesn’t have the complementary copies of the inverted repeat sequence with which it could form a double stranded stem with a loop.

11. General Discussion: is the presence of the origin next to the amplified region required for the formation of ODIRA-like events? The model requires the presence of the origin, and the events are called “origin-dependent”. However, is it possible that even without replication the fragment can still integrate and lead to amplification (for example as STR-type of event)? It isn’t clear how the ODIRA intermediate could form if it did not contain an origin. We believe we have ruled out that the hairpin intermediate is produced by a double stranded break that folds upon itself after enzymatic processing. If the hairpin fails to replicate in the next cell cycle and were to recombine with one of the replicated copies of chromosome II then it would just result in swapping of the telomeres between the two molecules. If there were a dogbone intermediate that recombined with a copy of chromosome II then the reciprocal products would be linears, both capped with a hairpin. In the following cell cycles, the centromere proximal product would undergo BFB cycles and the telomere proximal product would be lost since it has no centromere. In our 2015 paper (Brewer BJ et al. PMID 26700858) we show that when we deleted the origin adjacent to *SUL1* all of the inverted amplicons contain the next origin of replication. The origin is needed to create the hairpin/dogbone and to maintain the intermediates until they recombine with the chromosome.
